# First complete mitochondrial genome of *Pselliophora* (Diptera, Tipulidae): genome description and phylogenetic implications

**DOI:** 10.1080/23802359.2024.2381817

**Published:** 2024-07-19

**Authors:** Lei Zhang, Yalin Huang, Yunpeng Gai, Senlin Hou, Qingbin Zhan

**Affiliations:** a Department of Public Safety, Nanjing Police University, Nanjing, China; b Department of Criminal Science and Technology, Nanjing Police University, Nanjing, China; c Key Laboratory of State Forestry and Grassland Administration on Wildlife Evidence Technology, Nanjing, China; d School of Grassland Science, Beijing Forestry University, Beijing, China

**Keywords:** Crane fly, mitochondrial genome, *Pselliophora bifascipennis*

## Abstract

*Pselliophora* is widely distributed in Eurasia and China. To explore the characteristics of the mitogenome of *Pselliophora* and reveal phylogenetic relationships, the mitogenome of *Pselliophora bifascipennis* Brunetti, 1911 was sequenced and annotated. This is the first complete mitochondrial genome in this genus. Its mitogenome is 15821 bp in length, containing 13 protein-coding genes, 22 tRNA and 2 rRNA genes. Nucleotide compositions of its whole mitogenome are 39.09% for A, 38.49% for T, 13.42% for C, and 9.01% for G. Consistent with previous observations of Tipulidae species, the mitogenome of *Pselliophora bifascipennis* is highly conserved in gene size, organization and codon usage, and secondary structures of tRNAs. Most tRNAs have the typical clover-leaf structure. The control region is 1006 bp long with an A + T content of 92.7%. Phylogenetic tree analysis using the sequences of the mitochondrial genomes of *Pselliophora bifascipennis* and other Tipulidae species showed that *Pselliophora bifascipennis* is closely related to *Tanyptera hebeiensis*. These two species are grouped on the same branch, which is in accordance with the traditional morphological classification. The results of this study lay a foundation for screening molecular markers of mitochondrion for molecular identification and genetic structure research in Tipulidae species.

## Introduction

Tipulidae is a family of large crane flies classified within the order Diptera. With an extensive representation of over 30 genera and a total of 4,200 documented species, Tipulidae stands as a widely distributed and prevalent family of crane flies (Oosterbroek et al. 2020). One distinctive feature characteristic of Tipulidae crane flies is the measurement of their maxillary palps, a pair of appendages suspended from the anterior aspect of their head. The diagnostic criterion is met if the fourth segment of the maxillary palp, located farthest from the body, exceeds the combined length of the other three segments, serving as an indicator of Tipulidae membership. In larvae, a retractible hemicephalous head capsule can be a distinguishing feature (Pritchard 1983). Large crane flies typically have 13 antennal segments, whereas common limoniid crane flies usually have 14 or 16 segments. Their life cycle includes a short egg stage lasting 1–2 weeks, followed by four larval stages and a brief pupal stage of 1-2 weeks before short-lived adults emerge (Liu et al. 2010). Seasonal diapause, both in summer and winter, is frequently observed during specific stages, and the overall duration of the cycle can vary considerably, ranging from as short as 6 weeks to as long as 6 years, contingent upon the species and prevailing environmental conditions (Oosterbroek 1989).

The genus *Pselliophora*, a constituent of the family *Tipulidae* established by Osten Sacken in 1887, is distinguished by its type species, *P. laeta* (Fabricius) (Osten Sacken 1887). Within the genus *Pselliophora*, there are currently 108 recognized and valid species (http://ccw.naturalis.nl/ (Oosterbroek, 2024)). As of now, GenBank contains only 11 complete mitochondrial genomes for Tipulidae. The absence of mitogenome data for *Pselliophora* presents a significant obstacle to conducting a comprehensive phylogenetic analysis. In the scope of this study, we embarked on the task of sequencing and analyzing the inaugural complete mitochondrial genome of *Pselliophora*. Furthermore, phylogenetic analyses were undertaken to ascertain the phylogenetic placement of *Pselliophora bifascipennis*, contributing valuable insights into the evolution of mitogenomes and the broader phylogeny of *Tipulidae*.

## Materials

**Figure 1.**
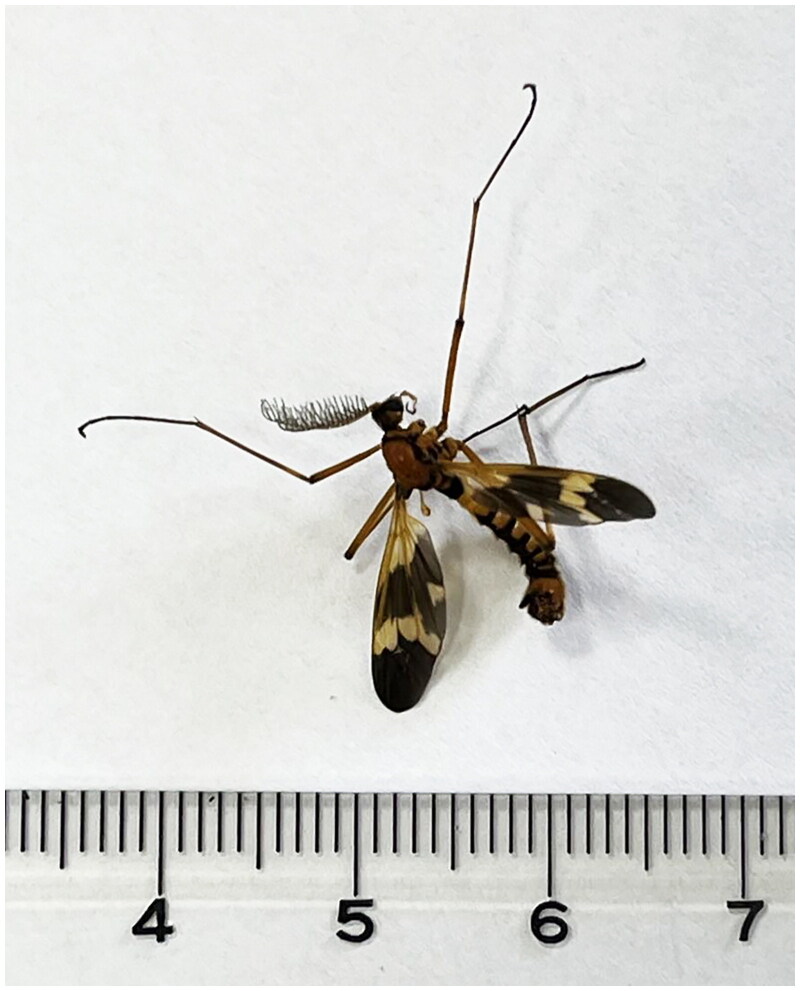
Morphological photograph of *Pselliophora bifascipennis* (photographed by QB ZHAN).

The material of *Pselliophora bifascipennis* (Figure 1) was collected from Nanjing, (Geographic location: 32°5'53.7072"N, 118° 48'33.8" E) Jiangsu Province, China, on 30 Sep. 2021. The sample was alive during the collection and the specimen was deposited in the Museum of Nanjing Police University under the voucher number NFPC0809 (Qingbin Zhan, zhanqb@nfpc.edu.cn). Species identification was carried out one by one under a stereomicroscope. And this species was identified with assistance from Dr. Yan Li of Shenyang Agricultural University.

## Methods

We extracted total genomic DNA from the chest muscles of adult using the cetyltrimethylammonium bromide (CTAB) method, as described by Shahjahan et al. (1995). We used 1 mg of DNA for preparing DNA libraries. These libraries were then sequenced using 150 bp paired-end reads on the Illumina NovaSeq 6000 platform (Illumina, San Diego, CA). Data quality control was performed with Fastp, according to Chen et al. (2018).

In total, 21,787,124 clean reads were generated. Quality control measures were implemented as follows: first, reads containing more than 10% unrecognized nucleotides were discarded. Second, reads with over 50% of bases possessing a Phred quality score below 5 were removed, along with any reads that exhibited alignment with adapters greater than 10 nucleotides in length, with allowances for mismatches ≤ 10%. Finally, potential PCR repeats generated during library construction, such as identical reads 1 and 2 in the paired-end readings, were eliminated. The mitochondrial genome of *P. bifascipennis* was assembled with Novoplasty v2.7, as described by Nicolas et al. (2016). We annotated the complete mitochondrial genome using the MITOS web server, following the methodology of Bernt et al. (2013). The mitochondrial genome map was created using Proksee, according to Grant et al. (2023).

To explore the phylogenetic position of *P. bifascipennis*, we analyzed 20 species sequences from the Dipetra group sourced from NCBI, designating *Sterphus plagiatus*, *Psilota atra*, and *Ocyptamus norina* as outgroup species. Recognizing the rapid evolutionary pace of the third codon position in protein-coding genes (PCGs) which can lead to substitution saturation and potentially complicate phylogenetic reconstruction (Breinholt and Kawahara 2013), we opted for caution in our analysis. The protein-coding sequences were aligned using MAFFT v7.505 (Katoh and Standley 2013), employing the '–auto’ strategy and codon alignment mode. To ensure clarity in our dataset, we eliminated gaps and ambiguous sites with trimAI (Capella-Gutiérrez et al. 2009), then concatenated into a PCG12RNA matrix (only the first and second codon positions of 13 protein-coding genes and two rRNAs) using PhyloSuite version 1.2.3 (Zhang et al. 2020; Xiang et al. 2023). Our phylogenetic analysis was based on the PCG12RNA matrix and we constructed maximum likelihood phylogenetic trees using IQ-TREE v2.2.0 (Nguyen et al. 2015) under the Edge-linked partition model, incorporating 5000 ultrafast bootstraps (Minh et al. 2013) and the Shimodaira-Hasegawa-like approximate likelihood-ratio test (Guindon et al. 2010). Additionally, Bayesian Inference phylogenies were generated with MrBayes v3.2.7a (Ronquist et al. 2012), applying a partition model over 2 parallel runs for 392,000 generations, discarding the initial 25% of sampled data as burn-in. We visualized and annotated the resultant phylogenetic trees using the Interactive Tree of Life (ITOL) (https://itol.embl.de/).

## Results and discussion

**Figure 2.**
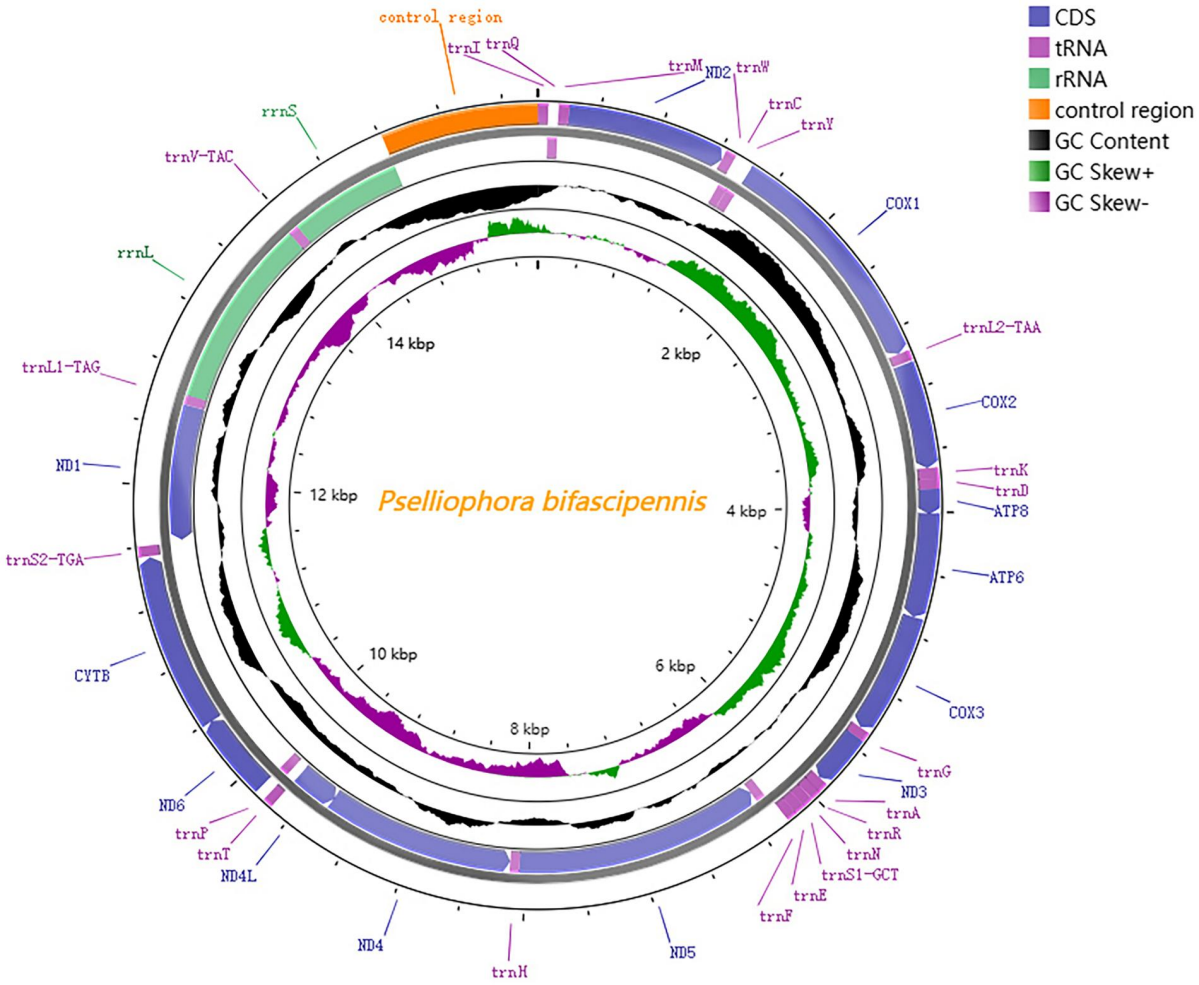
Circular map of the mitochondrial genome of *Pselliophora bifascipennis.* Genes outside the circle are encoded on the heavy strand and genes inside the circle are encoded on the light stand.

**Figure 3.**
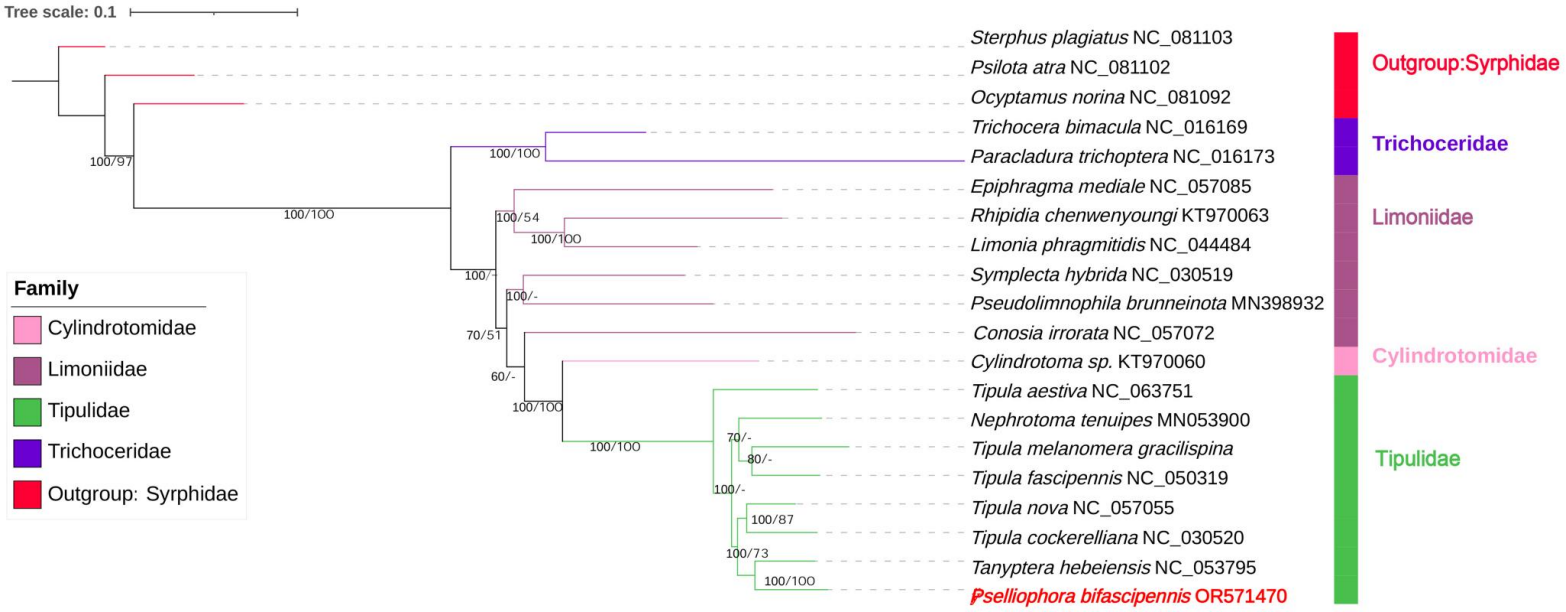
Based on the PCG12RNA matrix, the phylogenetic tree was reconstructed under both bayesian inference (BI) and maximum-likelihood (ML) methods, with branch support values denoted posterior probabilities and bootstrap values. The mitochondrial genomes of 20 species of Diptera and three outgroup species. *Trichocera bimacula* (Beckenbach 2012), *Paraclacura trichoptera* (Beckenbach 2012), *Limonia phragmitidis* (Ren et al. 2019), *Epiphragma mediale* (Zhang et al. 2021), *Symplecta hybrida* (Zhang et al. 2016), *Pseudolimnophila brunneinota* (Ren et al. 2019), *Conosia irrorata* (Zhang et al. 2019). *Cylindrotoma sp.* (Zhang et al. 2016), *Pselliophora bifascipennis* (OR571470), *Tanyptera hebeiensis* (Zhao et al. 2021), *Tipula nova* (Zhao et al. 2019), *Tipula fascipennis*, *Tipula aestiva* (Gao et al. 2023), *Tipula melanomera gracilispina* (Zhang et al. 2019), *Tipula cockerelliana* (Zhang et al. 2016), and *Nephrotoma tenuipes* (Ren et al. 2019).

The complete mitochondrial genome of *P. bifascipennis* (GenBank accession number OR571470) is 15,821bp in length and contains thirteen protein-coding genes (PCGs), twenty-two transfer RNA genes (tRNAs), two ribosomal RNA genes (rRNAs) (Figure 2). The mitogenome sequence has a high AT content of 77.58% (A: 39.09%, T: 38.49%). There are a total of 32 overlapped nucleotides between genes in 10 locations, ranging from 1 to 8 bp in length; while there are totally 69 bp intergenic nucleotides in 10 locations, ranging from 1 to 21 bp in length. Except *COX1* and *ND5* beginning with TCG and GTG codon, respectively, 11 PCGs started with ATN codons (*ND2, ATP8, ND3, ND6* with ATT, *COX2*, *ATP6*, *COX3*, *ND4*, *ND4L*, *CYTB* with ATG, *ND1* with ATA, nine PCGs terminated with a complete codon (*COX1*, *ATP8*, *ATP6*, *COX3*, *ND4L*, *ND6, CYTB*, *ND1* with TAA; however, *ND2*, *COX2*, *ND3*, *ND5*, *ND4* ended with an incomplete codon (with T) (Table 1). tRNAs had lengths from 64 bp to 72 bp, and could be folded in a typical cloverleaf structure, except the *trnF*, which lacked a dihydrouridine arm that had been simplified to a loop. The control region is located between srRNA and *tRNA^gau^* and is 1006 bp in length with an A + T content of 92.7%, which is the most AT-rich region of this mitogenome. The A + T content of the whole genome, PCGs, tRNAs, and rRNAs was 77.6%, 76.9%, 77.5%, and 79.4%, respectively. To understand the evolutionary status of *P. bifascipennis*, the mitochondrial genome of 20 species of Diptera and 3 outgroup species were used for phylogeny construction.In the maximum likelihood-based phylogenetic tree, *Pselliophora bifascipennis* is classified within the Tipulidae family, a significant component of the Tipuloidea superfamily. This family, along with Cylindrotomidae, Limoniidae, and Trichoceridae, collectively forms the diverse group within the Tipuloidea. *Pselliophora bifascipennis* is most closely related to *Tanyptera hebeiensis,* with 100% bootstrap support, as depicted in Figure 3. Additionally, it forms a group with *Tipula nova* and *Tipula cockerelliana,* indicating a close genetic relationship among these species.

**Table 1.** Mitochondrial genome characteristics of *Pselliophora bifascipennis.*

Gene	Location	Size (bp)	Strand	Start codon	Stop codon	Intergenic length
*trnI(gau)*	1-67	67	+			0
*trnQ(uug)*	65-133	69	–			−3
*trnM(cau)*	133-201	69	+			−1
*ND2*	202-1231	1030	+	ATT	T--	0
*trnW(uca)*	1232-1300	69	+			0
*trnC(gca)*	1293-1356	64	–			−8
*trnY(gua)*	1358-1423	66	–			1
*COX1*	1422-2957	1536	+	TCG	TAA	−2
*trnL(uaa)*	2958-3021	64	+			0
*COX2*	3032-3716	685	+	ATG	T--	10
*trnK(cuu)*	3717-3787	71	+			0
*trnD(guc)*	3787-3852	66	+			−1
*ATP8*	3853-4014	162	+	ATT	TAA	0
*ATP6*	4008-4685	678	+	ATG	TAA	−7
*COX3*	4685-5473	789	+	ATG	TAA	−1
*trnG(ucc)*	5476-5539	64	+			2
*ND3*	5540-5891	352	+	ATT	T--	0
*trnA(ugc)*	5892-5956	65	+			0
*trnR(ucg)*	5956-6019	64	+			−1
*trnN(guu)*	6022-6088	67	+			2
*trnS(gcu)*	6089-6155	67	+			0
*trnE(uuc)*	6156-6220	65	+			0
*trnF(gaa)*	6242-6307	66	–			21
*ND5*	6308-8039	1732	–	GTG	T--	0
*trnH(gug)*	8040-8105	66	–			0
*ND4*	8106-9441	1336	–	ATG	T--	0
*ND4L*	9435-9731	297	–	ATG	TAA	−7
*trnT(ugu)*	9734-9799	66	+			2
*trnP(ugg)*	9800-9865	66	–			0
*ND6*	9868-10395	528	+	ATT	TAA	2
*CYTB*	10395-11531	1137	+	ATG	TAA	−1
*trnS(uga)*	11539-11606	68	+			7
*ND1*	11625-12566	942	–	ATA	TAA	18
*trnL(uag)*	12571-12634	64	–			4
*l-rRNA*	12635-13957	1323	–			0
*trnV(uac)*	13958-14029	72	–			0
*s-rRNA*	14030-14815	786	–			0
D-loop	14816-15821	1006	+			0

## Conclusion

In this study, we reported the complete mitogenome of Pselliophora bifascipennis, which was 15,821 bp in length, containing 13 PCGs, 22 tRNA, two rRNA, and one D-loop region as a typical mitogenome. The phylogenetic tree we constructed showed these species were divided in to four clades, Limoniidae, Tipulidae, Trichoceridae, Cylindrotomidae. *Pselliophora bifascipennis* was shown as a sister taxon to *Tanyptera hebeiensis*. This is the first complete mitochondrial genome has been generated in this genus *Pselliophora*. Our works provided the phylogenetic information of Dipteran at the mitochondrial genome level for inferring the phylogenetic relationship of Dipteran species.

## Supplementary Material

sequencing coverage.png

## Data Availability

The data that support the findings of this study are openly available in GenBank at https://www.ncbi.nlm.nih.gov/genbank/, reference number OR571470. The associated BioProject, Bio-Sample numbers, and SRA are PRJNA1054234, SAMN38912920, and SRR27276545, respectively.
