# Supplementary figures and images for: First complete mitochondrial genome of Pselliophora (Diptera, Tipulidae): genome description and phylogenetic implications
Source: Mitochondrial DNA B Resour. 2024 Jul 19;9(7):897–901. doi: 10.1080/23802359.2024.2381817 (PMC11262202; doi:10.1080/23802359.2024.2381817)

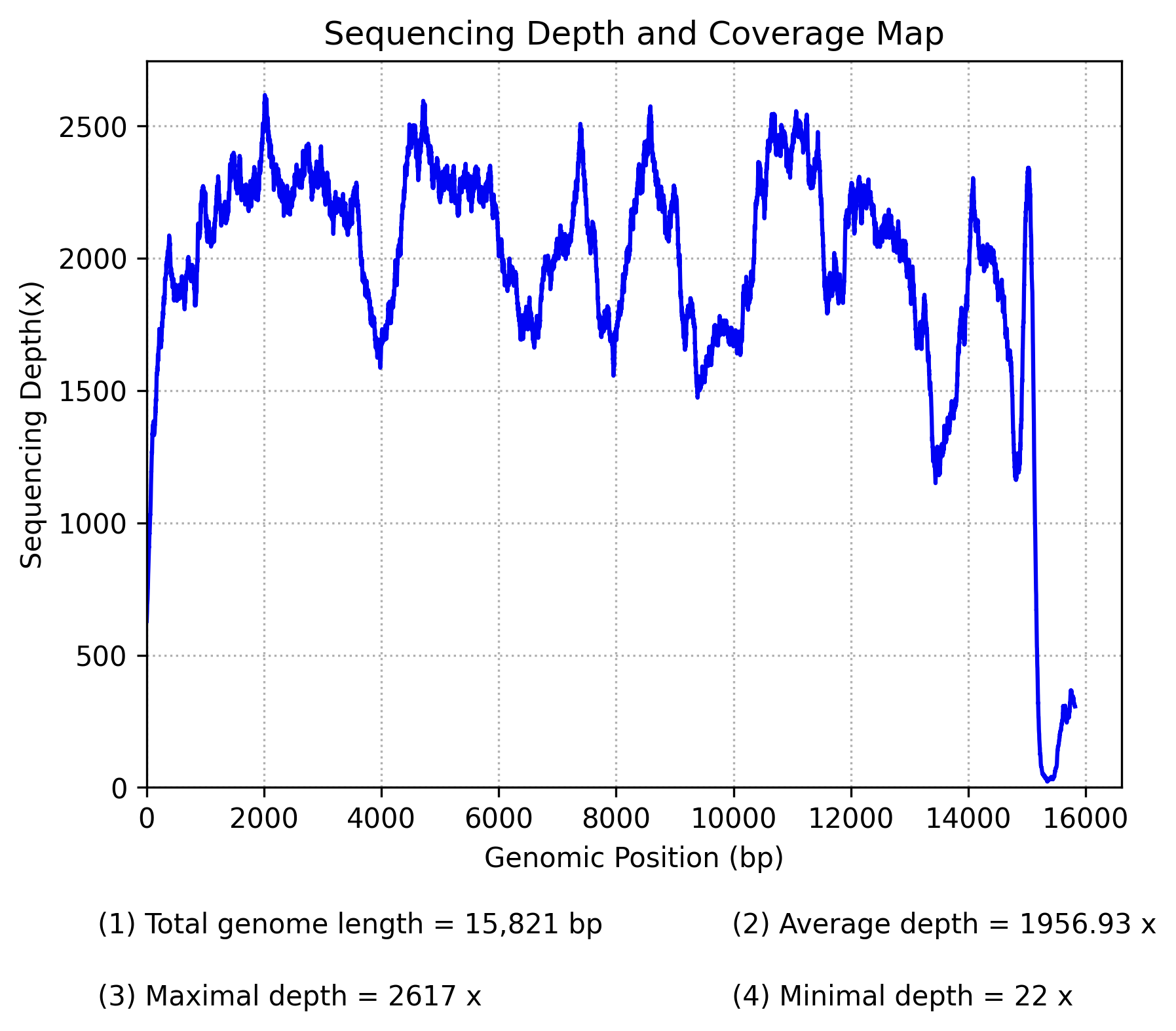

Supplement: sequencing coverage.png [file TMDN_A_2381817_SM8958.png]
